# ARGem: a new metagenomics pipeline for antibiotic resistance genes: metadata, analysis, and visualization

**DOI:** 10.3389/fgene.2023.1219297

**Published:** 2023-09-15

**Authors:** Xiao Liang, Jingyi Zhang, Yoonjin Kim, Josh Ho, Kevin Liu, Ishi Keenum, Suraj Gupta, Benjamin Davis, Shannon L. Hepp, Liqing Zhang, Kang Xia, Katharine F. Knowlton, Jingqiu Liao, Peter J. Vikesland, Amy Pruden, Lenwood S. Heath

**Affiliations:** ^1^ Department of Computer Science, Virginia Polytechnic Institute and State University, Blacksburg, VA, United States; ^2^ Department of Civil and Environmental Engineering, Virginia Polytechnic Institute and State University, Blacksburg, VA, United States; ^3^ Interdisciplinary PhD Program in Genetics, Bioinformatics, and Computational Biology, Virginia Polytechnic Institute and State University, Blacksburg, VA, United States; ^4^ School of Plant and Environmental Science, Virginia Polytechnic Institute and State University, Blacksburg, VA, United States; ^5^ Department of Dairy Science, Virginia Polytechnic Institute and State University, Blacksburg, VaA, United States

**Keywords:** antibiotic resistance genes, workflow, metagenomics, bioinformatics, genome annotation

## Abstract

Antibiotic resistance is of crucial interest to both human and animal medicine. It has been recognized that increased environmental monitoring of antibiotic resistance is needed. Metagenomic DNA sequencing is becoming an attractive method to profile antibiotic resistance genes (ARGs), including a special focus on pathogens. A number of computational pipelines are available and under development to support environmental ARG monitoring; the pipeline we present here is promising for general adoption for the purpose of harmonized global monitoring. Specifically, ARGem is a user-friendly pipeline that provides full-service analysis, from the initial DNA short reads to the final visualization of results. The capture of extensive metadata is also facilitated to support comparability across projects and broader monitoring goals. The ARGem pipeline offers efficient analysis of a modest number of samples along with affordable computational components, though the throughput could be increased through cloud resources, based on the user’s configuration. The pipeline components were carefully assessed and selected to satisfy tradeoffs, balancing efficiency and flexibility. It was essential to provide a step to perform short read assembly in a reasonable time frame to ensure accurate annotation of identified ARGs. Comprehensive ARG and mobile genetic element databases are included in ARGem for annotation support. ARGem further includes an expandable set of analysis tools that include statistical and network analysis and supports various useful visualization techniques, including Cytoscape visualization of co-occurrence and correlation networks. The performance and flexibility of the ARGem pipeline is demonstrated with analysis of aquatic metagenomes. The pipeline is freely available at https://github.com/xlxlxlx/ARGem.

## 1 Introduction

Antibiotic resistance poses a significant risk to human health. Antibiotic resistance genes (ARGs) encode resistance to antibiotics and can be carried in the bacterial chromosome or on mobile genetic elements (MGEs). ARGs are of greatest concern to human health, especially when they are found in known or emerging pathogens (Vikesland et al., 2019). The need for monitoring of ARGs in the environment, including water resources and agricultural production systems, is increasingly being recognized. Such environments play an important ecological role in propagation of ARGs. The ARGs can emanate from anthropogenic sources or from natural environments themselves, serving as facilitators of horizontal gene transfer (HGT) (Maiden, 1998; Barlow, 2009; Aminov, 2011; Lerminiaux and Cameron, 2019). HGT can contribute to expansion of the general reservoir of ARGs carried across environmental microbiomes, influencing human and animal pathogens, inducing new mechanisms of antimicrobial resistance. Metagenomics, the study of DNA extracted across the microbial community representing the environment of interest, has arisen as a promising approach to profiling ARGs and other microbial entities of concern, such as human pathogens (Koonin, 2018; Chiu and Miller, 2019). Environmental metagenomics has shown promise for tracking shifts in ARG and pathogen markers in the environment with time and in response to various disturbances and inputs (Berglund et al., 2019; de Abreu et al., 2021). Thus, metagenomics is being proposed as an efficient means of comprehensive surveillance of ARGs and pathogens across the One Health spectrum (Shen et al., 2021).

Contemporary environmental metagenomic data sets typically consist of a number of short read sequence files, typically generated by Illumina sequencing producing files ranging in size up to 100 Gbp (Gigabase pairs) (Davis et al., 2023), each representing either a Processing such datasets requires significant computational analysis. This typically needs to be organized in a bioinformatics pipeline that consists of selected software tools, which are mutually connected custom scripts. These scripts are usually written in programming languages such as Python 3 (Siegwald et al., 2017; Breitwieser et al., 2019), and composing such scripts to construct a bioinformatics pipeline can be challenging for non-expert users.

Many metagenomic analysis pipelines exist with much variation. However, the goal of a typical pipeline is to identify microbial taxa and genes of interest in a subset of samples, and to estimate their abundances. Further analysis of the annotation is often left to specific tools selected by the researcher. A pipeline may assemble the reads into contigs to allow identification of complete or nearly complete genes and to improve resolution for annotation. A classic metagenomics pipeline is the MG-RAST server, which is designed to process numerous samples on high-performance computing clusters (Meyer et al., 2008). A number of more recent pipelines (which we briefly review here) are available for a researcher to install and execute on their own computational resources (Uritskiy et al., 2018; Clarke et al., 2019; Dong and Strous, 2019; Tamames and Puente-Sanchez, 2019; Eng et al., 2020; Grieb et al., 2020). MetaWRAP employs binning and reassembly steps to obtain improved annotation (Uritskiy et al., 2018). SqueezeMeta concentrates on simultaneously assembling multiple metagenome data sets along with binning to enhance the identification of low-abundance taxa and genes (Tamames and Puente-Sanchez, 2019). MetaErg provides graphical summaries of the annotated contigs to support visual confirmation of contig quality (Dong and Strous, 2019). Sunbeam emphasizes a flexible pipeline framework that, in typical use, does not require the researcher to provide extensive run-time parameters (Clarke et al., 2019). Grieb et al. (Grieb et al., 2020) developed a pipeline explicitly tailored for research on marine plankton. Finally, MetaLAFFA is a flexible metagenomic analysis pipeline targeted to distributed computing environments (Eng et al., 2020).

A common limitation among the pipelines is a lack of integrated tools for additional analysis and visualization beyond basic annotation. Moreover, these pipelines do not provide flexible input, which results in a disincentive to data sharing and greatly detracts from the overall utility of the data. Metadata, which is the data describing properties (e.g., DNA extraction method and sample environment) of the sample, is nowadays commonly provided along with the sample sequences. Lack of extensive provision and sharing of metadata diminishes the ability to perform analyses that harness the power of metadata to support predictive modeling of environmental metagenomes. This deficiency in metadata sharing also detracts from encouraging reporting of comparable data, which is a critical need for the broader goal of large-scale environmental ARG monitoring. While researchers might recognize the importance of the extensive metadata that they collect for each sample, the actual types of metadata captured can vary greatly across research projects (Goncalves and Musen, 2019; Martinez-Romero et al., 2019). As one effort to remedy the situation, the National Center for Biotechnology Information (NCBI) (Sayers et al., 2019) collects a set of required metadata for each sample uploaded to resources, such as BioProject and BioSample (Federhen et al., 2014; Martinez-Romero et al., 2019), while still allowing for flexible column addition and following the minimum information about any (x) sequence (MIxS) guidelines (Yilmaz et al., 2011). However, comparing data across different projects remains a challenging task when using NCBI metadata.

Another notable framework, not limited to metagenomics analysis, is Galaxy (Jalili et al., 2020). Galaxy is a platform developed for flexible workflows that can be customized for bioinformatics tasks, with an open-source framework available for customization. Several pipelines have been developed using the Galaxy framework for various metagenomics tasks (Pilalis et al., 2012; Yang et al., 2016; Batut et al., 2018). Among them, only a few have aimed to develop an integrated pipeline that performs tasks beyond annotation. Additionally, most of these pipelines were not specifically designed for ARG detection tasks or for addressing the issue of customizing metadata in different environments.

Towards addressing the aforementioned issues, we present ARGem pipeline. This locally deployable pipeline supports ARG annotation as well as the capture of a flexible set of metadata, which will encourage comprehensive data sharing and be ultimately accessible to support more sophisticated future analysis after annotation is complete. To achieve this purpose, users are provided with a simple spreadsheet with required and recommended metadata fields and standardized units. Users complete the spreadsheet and submit it as input to create an ARGem project, in which the data are stored in a relational database that can be further cross-analyzed.

Key analytical tools and capabilities that are commonly applied for metagenomic-based ARG monitoring have been built into the ARGem pipeline, extending data analysis beyond the annotation of taxa and ARGs to include statistical analysis and ARG co-occurrence and correlation networks. The resulting outputs can culminate in a wide range of custom visualizations to support comparisons across samples and projects, as well as tables summarizing the results in tabulated format to support additional analysis. As detailed in Section *Assembly and Annotation*, we have extensively examined the bioinformatics components of the ARGem pipeline. In particular, we prioritized comprehensive databases for ARGs and MGEs annotation. One comparable pipeline is our own MetaStorm server (Arango-Argoty et al., 2016), which is only available as a Web service to execute on the computational resources of an individual research lab, which allows extendability of ARGem with new capabilities. PathoFact (de Nies et al., 2021) is a resource specialized in the prediction of ARGs and pathogens and make uses of our DeepARG resource (Arango-Argoty et al., 2018). However, PathoFact does not have the flexibility to incorporate or update reference databases other than the provided options, which were released prior to 2021. Also, PathoFact does not handle the assembly step and requires pre-assembled contigs as the input, prioritizing post-assembly analysis rather than a full sequence-to-analysis pipeline. PathoFact depends on Miniconda to guaranteee compatibility with specific versions of Snakemake and Python, making it convenient for users to install and use at the time of release, but may later lead to obsolescence compared to software with such dependency.

Overall, ARGem is a locally deployable pipeline which addresses many of the needs identified above through a user-friendly, full-service pipeline for ARG analysis of environmental metagenomic data with enhanced metadata capture and normalization to facilitate comparison within and across studies. In the *Method* section, we describe in detail the tools and methods employed in the ARGem pipeline. In Section *Results*, we describe the overall workflow of the pipeline and the general mechanism for each step, as well as demonstrate the value of our ARGem pipeline with a number of example runs utilizing metagenomic samples relevant to aquatic environments. Sections *Discussion* and *Conclusion* emphasize the strengths of our current implementation and identify potential paths for future extensions.

## 2 Methods

The ARGem pipeline integrates a number of tools implemented as individual modules that can be used within the pipeline or independently. Detailed descriptions are included for task scheduling, the Luigi workflow builder (Luigi Development Team, 2020), data retrieval, reference databases for annotations, assembly and annotation, analysis, visualization and the relational database.

### 2.1 Task scheduling

The ARGem pipeline consists of a sequence of tasks and employs a task scheduling mechanism that handles the distributed resources on multiple servers. This scheduling strategy is adequate for the computational resources of a typical lab. By maintaining a straightforward and concise task scheduling system, we intend to keep the system at lab scale and make it convenient for most researchers to use.

Specifically, we use the batch command in Linux. The batch command implements internal queues to manage and execute tasks in a manner that adapts execution demand to system capabilities, maintaining a ceiling on system load. If the job exits with an error, batch is used to catch the exception, and ARGem sends an email notification to the user email address stored in the database associated with the task. If the job completes successfully, the system also sends out an email notifying the user of the completion of the task.

### 2.2 Luigi workflow builder

Some of the tasks employed by ARGem are particularly time-demanding, such as sequence assembly and annotation. Such tasks can be especially demanding for analysis of environmental metagenomes, which tend to be particularly complex. In such cases, it is useful to incorporate a built-in workflow to handle the execution of tasks and deal with computational issues typically associated with long-running processes, such as error handling and status visualization. For ARGem, the Luigi package for Python (Luigi Development Team, 2020) is used by the back end to define tasks and chain them together to construct a workflow for the pipeline, as well as managing the scheduling of tasks, handling errors, and visualizing the status of the pipeline.

**FIGURE 1:**
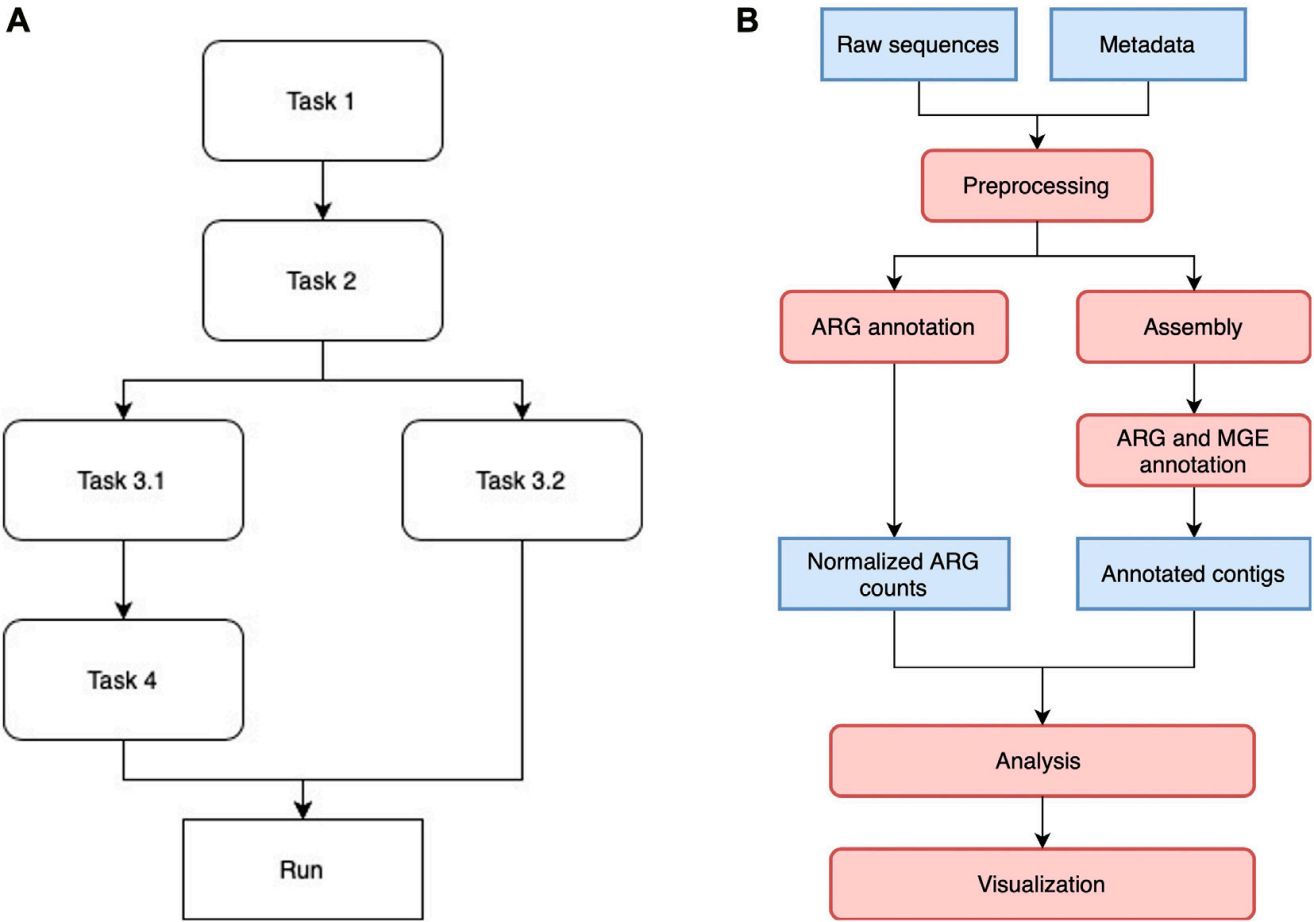
**(A)** A sample Luigi workflow. The workflow can consist of both linear and parallel tasks. A task that depends on a previous one will not be executed until all the dependencies have been completed. Tasks without direct or indirect dependency on each other can be executed in parallel if resource permits. **(B)** ARGem workflow. The ARGem pipeline automatically processes the raw sequences after a list of SRA accession numbers are submitted through a metadata spreadsheet. After preprocessing, the raw sequences go through two different branches: 1) short reads matching to generate normalized ARG counts, and 2) contig annotations against ARG and MGE reference databases. The results generated can then be passed to the integrated analysis and visualization tools. The default normalization methods built in the pipeline are 16S rRNA, TPM, and FPKM. 1) Blue rectangles indicate data and 2) red rounded rectangles indicate processing steps.

Luigi manages multiple tasks in the workflow by assigning them to different classes and drivers. Each class is designed to execute a particular task, such as short reads annotation or co-occurrence network analysis. Once the Luigi task classes are defined, they are aligned with each other in a workflow by indicating the dependencies between pairs of modules. Tasks without direct or indirect dependency on each other can be run in parallel, depending on how much resources the scheduler allocate for them. Figure 1A shows a generic Luigi workflow. In ARGem, all the Luigi modules are aligned linearly with a potential change on paralleling short read annotation with contig assembly and annotation, if needed.

### 2.3 Data retrieval from public websites

ARGem provides automatic raw sequence data retrieval from the public NCBI database (Sayers et al., 2022) through SRA toolkit (SRA Toolkit Development Team, 2022). The ARGem input spreadsheet contains an SRA number field in which the user can indicate the SRA or SRR number of the sample. The sample numbers then allow the SRA Toolkit to retrieve raw sequence data samples in *.fasta or *.fastq format. For the uploaded SRA numbers via the input spreadsheet, ARGem checks whether the SRA project numbers are accessible a suitable format through a pre-download. Invalid SRA numbers or those associated with incorrect formats are logged to a designated log file. Upon completion or interruption of the pipeline, these SRA numbers are then reported to the user in an email notification.

Once the accession and format verification is complete, ARGem begins the SRA sample retrieval process where the raw sequence files are downloaded individually for each SRA number. The retrieval of each sample is initialized with a query to the accession-size of the SRA project numbers through the SRA Toolkit to ensure that the size of a single sample is lower than the hard limit, which is set by default to be 70 gigabytes. In the case where the SRA sample is above the size limit, an error can be raised and logged accordingly by ARGem pipeline. Once the size verification is complete, the SRA sample is prefetched via the SRA Toolkit in *.sra format and then converted into *.fastq format. For paired end samples, the file format conversion process is split to convert each SRA sample into a paired files for assembly.

After all the raw sequence files are retrieved from NCBI website (Sayers et al., 2022), ARGem will initiate a post-download validation on the retrieved raw *.fastq sequence data files to validate their data integrity. Upon completion, annotation and assembly tasks for the valid samples will be scheduled according to the Luigi workflow.

### 2.4 ARG and MGE databases

The pipeline design of ARGem offers easy and flexible updates and interchanges for databases. Once a new ARG or MGE database is converted into a fasta file and a proper format for the annotation tool, ARGem redirects assembly and annotation tasks into the new databases. Default ARG and MGE databases were selected based on how widely they are used for metagenomic analysis, with a preference for databases that are frequently updated.

To annotate the raw sequences and assembly results into ARGs, ARGem integrates the current Comprehensive Antibiotic Resistance Database (CARD) (Alcock et al., 2020) as the default reference database, while the users have the option to use other databases at their choice.

ARGem utilizes three databases for MGEs: Mobile-OG (Brown et al., 2022), NanoARG (Arango-Argoty et al., 2019), and Parnanen et al. (Parnanen et al., 2018). The Mobile-OG database is a recently published database aiming to mitigate the high positive rates originated from accessory genes that are temporarily associated with the MGEs. The goal of the database is to provide high-quality annotations and annotations derived exclusively through bioinformatic evidence. NanoARG is a database that has been particularly insightful in identifyimg ARGs in sequences of varying lengths and a range of sequencing error rates. NanoARG is an integration of two data sets, NCBI and integron-integrase (I-VIP) database (Zhang et al., 2018). In the NanoARG database, MGE sequences have been extracted from NCBI using keywords such as “transposase,” “transposon,” “integrase,” “integron,” and “recombinase”, following the method described in (Forsberg et al., 2014). The I-VIP database focuses on comprehensive information on class 1 integrons. After extracting the MGE sequences from NCBI, the integrases of class 1 integrons have then been extracted from I-VIP database and added into the NanoARG database (Arango-Argoty et al., 2019). The Parnanen et al. MGE database (Parnanen et al., 2018) was created with a focus on mother-infant MGE sharing, providing a unique perspective and addition to the existing MGE research. This database was constructed by fetching coding sequences for genes that were annotated as IS*, ISCR*, intI1, int2, istA*, istB*, qacEdelta, tniA*, tniB*, tnpA* or Tn916 transposon open reading frames (ORFs). The genes were either sourced from the NCBI nucleotide database, or from the PlasmidFinder database (Carattoli and Hasman, 2020).

### 2.5 Assembly and annotation

The sequence data used in this study are available from the NCBI database (Sayers et al., 2022) and retrieved with the SRA Toolkit (SRA Toolkit Development Team, 2022) using the SRA accession numbers listed in the metadata table.

To select a suitable assembler for our short read metagenomic data, we carefully evaluated the performance of a set of assemblers on our server and on targeted data sets. The pre-selected set of assemblers was chosen based on evaluations in previous studies (Vollmers et al., 2017; Ayling et al., 2020; Zhang et al., 2020). Table 1 and Supplementary Table S1 summarizes the results of different analyses of these samples.

**TABLE 1:** An evaluation of assemblers on our server. In total one reclaimed waste water sample (water sample 1), one final treated biosolids sample (water sample 2), and two raw sewage and treated wastewater samples (water sample 3 and 4), were used to evaluate the performance of assemblers on our server. Note that the samples used here are different from those presented in Section *Results*. The size column shows the sizes of sample sequence files in gigabytes. Length indicates the sequence length of each sample sequence data. Time shows the total hours required to assemble the metagenomic data generated from a given sample. Percent of CPU, maximum resident set size and major page faults shows metrices reported by *time* command during the process.

Sample	Size (GB)	Length	Assembler	Time (hr)	Percent of CPU (%)	Maximum resident set size (KB)	Major page faults
Water1	5.91	108	MetaSPAdes	4 : 05: 43	1,147	46328252	31
Water1	5.91	108	IDBA-UD	2 : 47: 37	3130	32219196	1
Water1	5.91	108	MegaHIT	0 : 33: 47	3118	5369920	4
Water2	1.52	92	IDBA-UD	0 : 21: 20	2999	8617580	1
Water2	1.52	92	MegaHIT	0 : 05: 37	3109	1399316	1
Water2	1.52	92	MetaSPAdes	0 : 37: 23	1090	11654044	1
Water3	4.57	202	MegaHIT	0 : 43: 20	3402	4125444	0
Water3	4.57	202	MetaSPAdes	2 : 25: 05	1114	37923596	1
Water4	5.91	202	MegaHIT	0 : 54: 59	3,398	5014884	3
Water4	5.91	202	MetaSPAdes	3 : 13: 46	1,116	42655912	25

We evaluate the assemblers on the samples as follows: one reclaimed waste water sample (water sample 1), one final treated biosolids sample (water sample 2), and two raw sewage and treated wastewater samples (water sample 3 and 4) for the results depicted in Table 1. Note that the samples used here are different from those presented in Section *Results*. The first two samples were produced by our group, and the latter two samples were published in previous work (Lekunberri et al., 2018). For the first two wastewater samples we tested three assemblers: MetaSPAdes (Nurk et al., 2017), IDBA-UD (Peng et al., 2012) and MegaHIT (Li et al., 2015). While the annotation results of IDBA-UD and MegaHIT were similar, MegaHit showed a better performance in terms of time and memory usage in our test scenario. For the other two wastewater samples, we compared MetaSPAdes and MegaHIT. Overall, we found that on our data sets, MegaHIT generated reasonable results in a relatively short amount of time. Therefore we provide MegaHIT as the default assembler.

DIAMOND (Buchfink et al., 2015; 2021) was incorporated as the primary annotation tool across ARGem, both for short reads matching and contig annotation. DIAMOND is a open-source sensitive protein aligner used widely in the bioinformatics field. DIAMOND performs double-index alignment with a reduced alphabet and spaced seeds. DIAMOND has been reported to consume less amount of time for high-throughout scenarios compared to BLASTX (Camacho et al., 2009) and BLASTP in similar settings. We also use BLAST for our optional MGE Parnanen et al. (Parnanen et al., 2018) database for the nucleotide annotation, which is not available in DIAMOND.

### 2.6 Gene Co-occurrence and correlation analysis

Co-occurrence analysis is a widely applied technique in bioinformatics, and can infer important relationships among genes, such as their taxonomic host, their tendency to be co-expressed, and their ability to be co-mobilized via HGT (Faust and Raes, 2016). Sequencing depth is an important factor that influences the coverage and accuracy of assembly and thus the accuracy of co-occurrence analysis. This, in addition to inherent differences in microbiomes (diversity, representation in databases, etc.) creates difficulties for identifying a single method to accurately calculate gene correlations.

For co-occurrence analysis of ARGs and MGEs, the ARGem pipeline combines an ARG database and an MGE database to count the number of co-occurrence of contigs for each pair of one ARG and one MGE.

For correlation analysis, ARGem first imputes the missing values with zeros for the abundance data and then renormalizes it to be relative abundance data. This method is adapted from (Tao, 2014). We assume that the expression of each pair of genes is generated by an underlying bivariate normal distribution. Considering a gene pair denoted as (*x*_1_, *x*_2_), we calculate the mean values (*μ*_1_, *μ*_2_), the standard deviation (*σ*_1_, *σ*_2_), and the correlation *ρ*. To accomplish this, we need at least three complete gene pairs. Let *N* be the total number of experiments, and let *f*(⋅) represent the probability density function (pdf) of the underlying bivariate normal distribution. *F*(⋅) represents the combination of the pdf and the cumulative distribution function (cdf) of the normal distribution. The likelihood function *L* is defined as follows:

$$\begin{array}{ccc} L\left(\hat{\theta }|x_{1},x_{2}\right)= & & \\ & & \overset{N}{\underset{i=1}{\prod }}f{\left(x_{i1},x_{i2}\right)}^{\delta_{i1}\delta_{i2}}\cdot \frac{\partial }{\partial x_{1}}F{\left(x_{i1},c_{2}\right)}^{\delta_{i1}\left(1-\delta_{i2}\right)}\cdot \\ & & \frac{\partial }{\partial x_{2}}F{\left(c_{1},x_{i2}\right)}^{\left(1-\delta_{i1}\right)\delta_{i2}}\cdot F{\left(c_{1},c_{2}\right)}^{\left(1-\delta_{i1}\right)\left(1-\delta_{i2}\right)}, \end{array}$$

where *c*_1_ and *c*_2_ are the detection cut-offs for *x*_1_ and *x*_2_, and *δ*_*i*1_ and *δ*_*i*2_ are indicator variables indicating whether or not data is available for each *x*_*ij*_.

In the above equation, we first calculate the regular likelihood term *f*(⋅) when data are available for both pairs and then the second term factorizes into the pdf of *x*_1_ and the cdf of *x*_2_ at the cutoff term in a normal distribution that is shifted up by the distance of the current *x*_1_ observation from its mean multiplied by the correlation coefficient and scaled by the ratio of variances using *F*(⋅). If the correlation between the genes is strong, we expect the cdf of *x*_2_ at the cutoff to be directly related to the distance of *x*_1_ from its mean and *vice versa*. Then we calculate the joint cdf of the bivariate normal distribution at both cutoffs. The joint cdf term grows as the values of the cut-offs rise relative to their corresponding means. As this term increases, it tends to overshadow information from other terms.

Our approach involves maximizing the likelihood of observing a given expression pair while adjusting for a known cut-off threshold. In addition, we also capitalized on the data structure by introducing correlation bounds. To obtain sharper correlation estimates, we further utilize the partial correlation definition inequality to update our correlation estimates based on the correlation between other pairs. In this way, the proportional value of relative abundance can directly reflect the degree of correlation of the potential related gene pairs and we are able to produce correlation estimates even with severe missing data issues.

In the next step, our user can apply the desired threshold within the range [−1, 1] on the correlation matrix to filter out the relevant gene pairs for further analysis or visualization.

### 2.7 Visualization

Network analysis provides an intuitive means to visualize predicted relationships within bioinformatics fields, such as protein-protein interaction networks (Bharadwaj et al., 2017), gene-gene networks (Franz et al., 2016), and gene co-expression (Zhang and Horvath, 2005). ARGem approaches visualization from two perspectives: correlations and co-occurrences. Correlation graphs show relations among ARG annotation results without MGE using the method described in *Gene Co-occurrence and Correlation Analysis*. Co-occurrence graphs map ARGs and MGEs based on a number of co-occurrence pairs annotated on the same contigs assembled from raw sequences. For example, in three contigs *C*1, *C*2, and *C*3 in one sample, all contain the ARG-MGE pair ARG *A*1 and MGE *M*1, the occurrence of (*A*1, *M*1) is 3. The width of the edge between *A*1 and *M*1 will reflect the co-occurrence, in this case, which is 3. In correlation graphs, the width of the edges is based on the correlation score between two genes *Gene Co-occurrence and Correlation Analysis* and indicates the relative strength of the relationship in *Assembly and Annotation*. The size of each node is determined based on the sum of abundance in the metagenomic library. Co-occurrence networks, on the other hand, are an analysis of ARGs and MGE annotated on assembled contigs (1,000+ bps). Each edge that connects an ARG node and an MGE node represents the count of the given combination, where the width of the edges indicates the frequency that the combination is encountered (Arango-Argoty et al., 2019). Note that in co-occurrence networks, ARG nodes are only connected to MGE nodes.

ARGem by default builds correlation graphs and co-occurrence graphs using Cytoscape.js (Smoot et al., 2011; Franz et al., 2016), an open-source JavaScript-based graph library (Franz et al., 2016). Cytoscape provides interactive features so that users can select the target genes or filter the abundance rank from the network. Cytoscape library also enables changes in graphic scale, which can be adjusted to end users’ preferable size of visualized images. Other tools such as Python NetworkX library (Hagberg et al., 2008) are also included or can be made available for visualization.

### 2.8 Relational database

**FIGURE 2:**
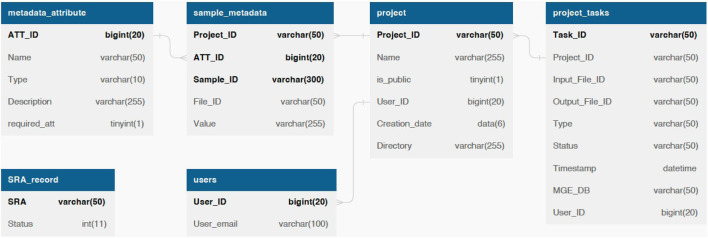
The database schema of ARGem. ARGem supports custom metadata attributes and various data processing parameters. Mandatory information including the SRA number and user information are reflected in database tables as *NOT NULL* fields. Optional fields are not required and can be set to a default value.

We employ the MySQL database for data management and storage. The database schema is shown in Figure 2. Only general information such as the SRA number and email address are required for data retrieval and task status notification. As for optional fields, we provide default data processing and visualization parameters, such as the MGE database and the co-occurrence threshold. Users can customize these parameters to meet their specific needs.

By allowing users to upload customized metadata spreadsheets, our database design can expand to include arbitrary metadata attributes. We record user custom metadata entries in the *metadata_attribute* table, which are available for all projects. With custom metadata, users can compare and visualize data across different projects. For an example metadata spreadsheet, see Supplementary Material.

## 3 Results

### 3.1 Pipeline

The ARGem pipeline consists of multiple computational components arranged primarily in a linear sequence, with built-in detection of certain error cases that serve to halt the pipeline early and send out an email notification of the error. We integrated the ARGem pipeline as a key component in the web-based platform AgroSeek (Liang et al., 2021). ARGem can also be deployed in other systems that incorporates a relational database management system, as detailed in Section *Relational Database*. The overall workflow is depicted in Figure 1B. For a more detailed workflow diagram, see the Supplementary Figure S1.

The typical pipeline steps are summarized in the following sub-sections.

#### 3.1.1 Input spreadsheet for a project

An ARGem Excel spreadsheet was designed through collaboration with environmental scientists to identify required *versus* recommended metadata for samples of various categories, along with specified reporting units. As an example, for aquatic environment samples, required metadata columns include the kind of experiment type from which the sample was collected (e.g., lab, field or pilot, selected from a drop-down menu), the DNA extraction method, the DNA sequencing platform, DNA sequencing output (e.g., single or paired reads), and the SRA accession number for each sample. The required columns are provided along with conditional columns depending on the type of aquatic environment matrix selected from the drop down menu.

Through an SRA number column, each sample is associated with a unique SRA number (Sayers et al., 2022) in the input spreadsheet. Therefore the raw data sequences can be conveniently retrieved from the online repository, if they have not yet been added to local data storage data. A complete, filled ARGem spreadsheet provides useful information on both the metadata and the raw data sequence, which can support richer analysis and visualization in later steps of the pipeline. In addition, a relational database associated with the pipeline is provided to store and manage the uploaded data, as well as the status of created projects.

Typically, the user selects one template from the ARGem library of spreadsheet templates that best represents the environment under study, customizes the template for their project, and enters the metadata into the spreadsheet with one row for each metagenomic sample.

There are in total six templates in the library, including five templates for different environment sample types and one user custom template. Through collaboration with environmental scientists, we designed specific templates for water, soil, treated or raw manure, pre- and post-harvest crop production system, and air samples.

#### 3.1.2 Retrieve DNA sequence data from NCBI

In this step, sequence data are retrieved based on the input SRA numbers provided for each sample in the metadata spreadsheet. These data subsequently serve as raw sequence files for the samples required for subsequent analysis.

#### 3.1.3 Assemble each DNA sample into contigs

In this step, the pipeline assembles the retrieved sequence files using the integrated assembly tool, namely, MEGAHIT (Li et al., 2015). This assembler was selected after evaluation on our server and targeted data sets. For details of the assembler evaluation, see Supplementary Table S1. The results of this step are a set of contigs for each sample.

#### 3.1.4 Annotate known ARGs and MGEs in short reads and contigs

This step performs annotation on both the assembled contigs (long-contig annotation) and retrieved short reads (short reads matching) using the integrated annotation tools (BLAST (Altschul et al., 1990) and DIAMOND (Buchfink et al., 2015; 2021)). The reference databases used for this step include an ARG reference database CARD (Alcock et al., 2020) along with three optional MGE reference databases: MobileOG (Brown et al., 2022), NanoARG (Arango-Argoty et al., 2019) (which is the database also used in our MetaCompare (Oh et al., 2018) service), and Parnanen et al. (Parnanen et al., 2018). The annotated genes for each sample are sent to output text files along with their relative abundances.

#### 3.1.5 Analysis

After obtaining the assembly and annotation results of each sample, the pipeline performs a set of analyses based on the results and the metadata attributes. Because it is not possible to discern ARGs imparted by mutations in housekeeping genes from true housekeeping genes, due to limitations in the resolution of sequencing technologies, ARGem excludes housekeeping genes from ARG analysis. A list of excluded genes is provided in the Supplementary Material. The results of the analysis are then made available to the users, usually in the form of tabular files. After this step, more optional analysis requiring user input parameters can be performed according to the desires of the user.

#### 3.1.6 Visualization

For the gene co-occurrence and correlation analysis results, corresponding visualizations are generated and provided to the users. Some of the visualizations can be customized by user-selected parameter inputs.

#### 3.1.7 Notification

After obtaining the results of each sample, or if the pipeline halts early, an email notification is sent to a designated e-mail address reporting the final status (success, partial success, or failure) of the pipeline. When the pipeline does not execute successfully, the notification will include specific information about the detected errors to help guide the user in addressing the problem.

### 3.2 Verification

The ARGem pipeline was tested using publicly-available data extracted from the NCBI database (Sayers et al., 2022). Results shown in this section are based on 15 fresh water samples obtained from BioProject PRJNA287840, collected monthly from 6 sites in 3 southwestern British Columbia streams over 14 months (Vlok et al., 2019). In the analysis results presented later, these 15 samples were arbitrarily divided into three groups to illustrate the functionality of the tools, rather than to reflect the inherent characteristics of the data. The results presented in this study have been annotated with one of the pipeline’s default MGE databases. However, users have the option to choose a different database or integrate their preferred database into the pipeline.

The pipeline generated tables that summarize results for three analyses: 1) short read matching to profile ARGs and estimate their relative abundances, 2) assembly of contigs from short reads, and 3) annotation of ARGs and MGEs in assembled contigs. Short read matching results for these fifteen samples yielded 380 annotated ARGs found in at least one sample out of the fifteen, with 16S rRNA, TPM and FPKM normalization reported in three separate files. Contig assembly generated assembled contigs for all fifteen samples. The ARG and MGE annotation based on assembled contigs generated one table of annotated ARGs and one table for annotated MGEs, for each sample. A table was also generated to report ARGs and MGEs that were found to co-occur in the samples.

**FIGURE 3:**
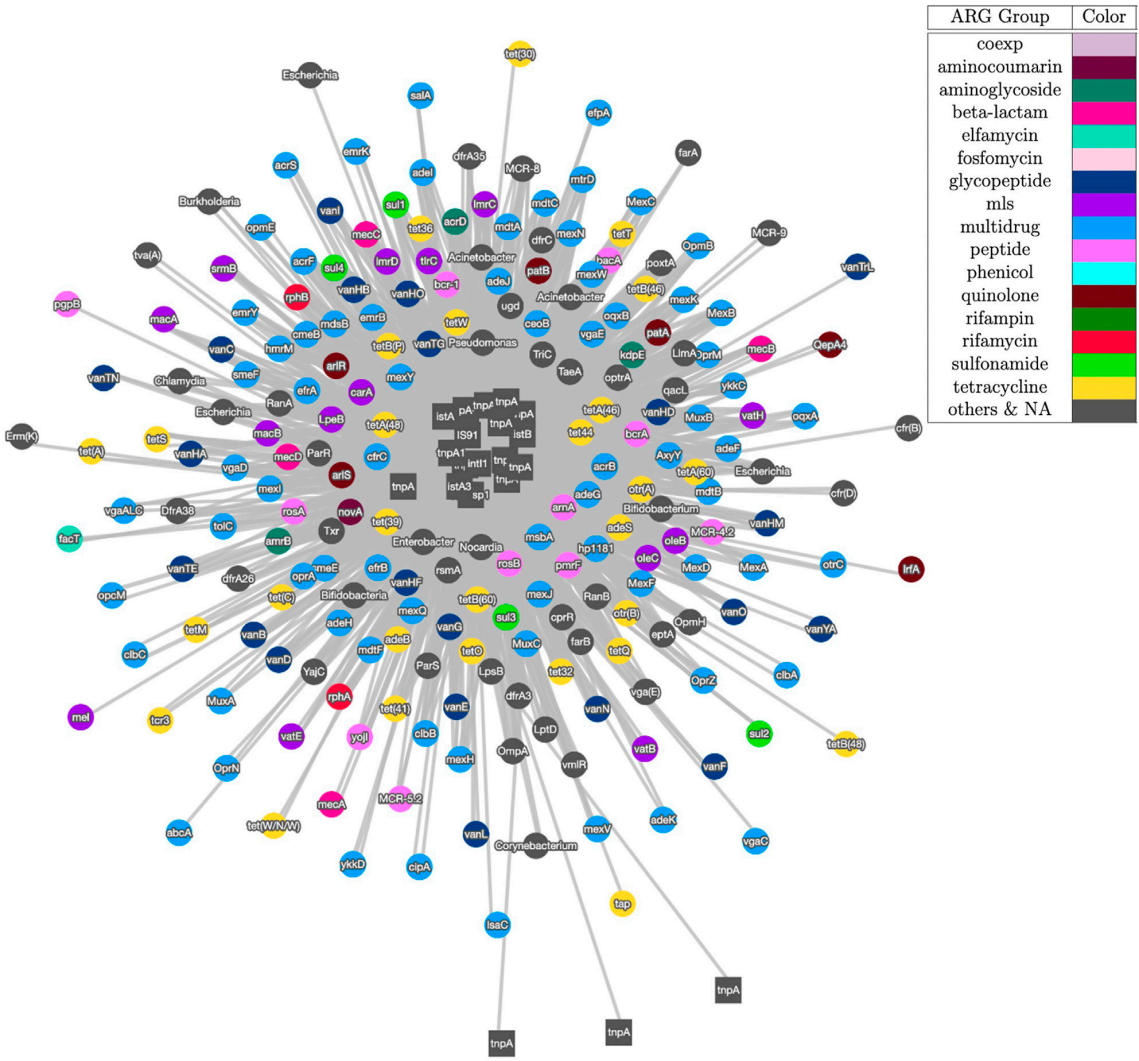
Co-occurrence graph generated using Cytoscape with threshold of 3 of samples SRR2088951, SRR2088982, SRR2088983, SRR2089011, SRR5571001, SRR5997542, SRR5997549, SRR9141345, SRR9141349, SRR9141356, SRR9141357, SRR9141362, SRR9141365, SRR9141380, and SRR9141383. MGEs are represented as square node and ARGs are shown as circle nodes. The colors of ARG nodes correspond to classification according to the corresponding class of antibiotic resistance assigned in CARD database (Alcock et al., 2020). The width of the edge between ARGs and MGEs in proportion to the number of common occurrences of each pair.

Figure 3 shows the visualization result based on contig assembly and annotation. This analysis and visualization is included in the ARGem pipeline. This is a co-occurrence network based on ARG and MGE annotation results on assembled contigs, using reference database CARD (Alcock et al., 2020) and Parnanen et al. (Parnanen et al., 2018), respectively. The co-occurrence graph is generated based on the number of co-occurrences in the sample. Once each combination of the MGE-ARG pair is counted, the pipeline filters the number of occurrences based on user input. Filtered pairs generate a co-occurrence graph, where nodes represent ARGs and MGEs detected and edges represent their occurrence together.

**FIGURE 4:**
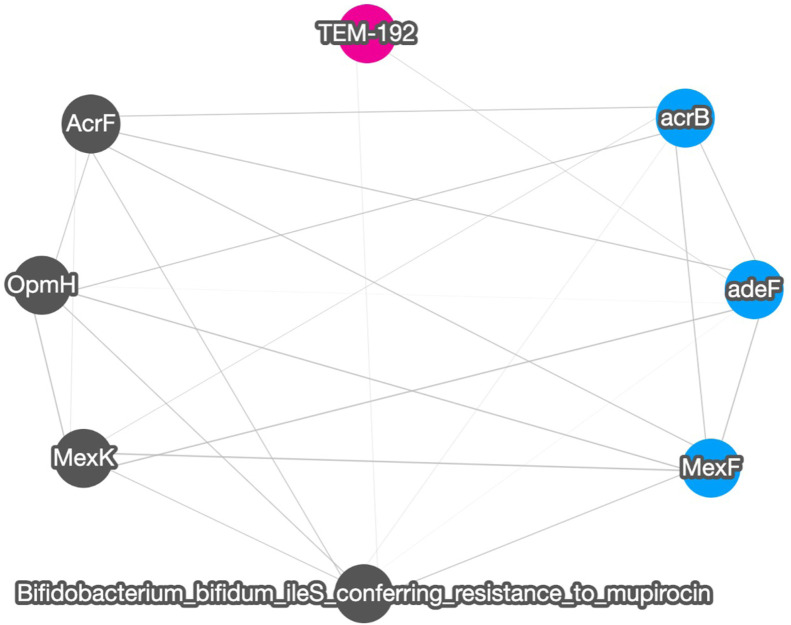
A correlation graph for 16S rRNA normalized short read matching result of samples SRR2088951, SRR2088982, SRR2088983, SRR2089011, SRR5571001, SRR5997542, SRR5997549, SRR9141345, SRR9141349, SRR9141356, SRR9141357, SRR9141362, SRR9141365, SRR9141380, and SRR9141383. The color codes are the same as in Figure 3. The width of the edge between ARGs and MGEs is in proportion to the absolute correlation value of each pair.

Figure 4 shows the correlation result based on short read matching. Given the 16S rRNA normalized ARG annotation generated by the pipeline, a correlation matrix was generated by the pipeline’s correlation analysis module and visualized as a correlation graph. The correlation matrix calculated by our proposed method reports a range from −1 to 1 and excludes single paired combinations, where only two data points or less were found. See also Supplementary Figure S2 for the correlation visualization output using Python NetworkX library instead of the default option Cytoscape.

**FIGURE 5:**
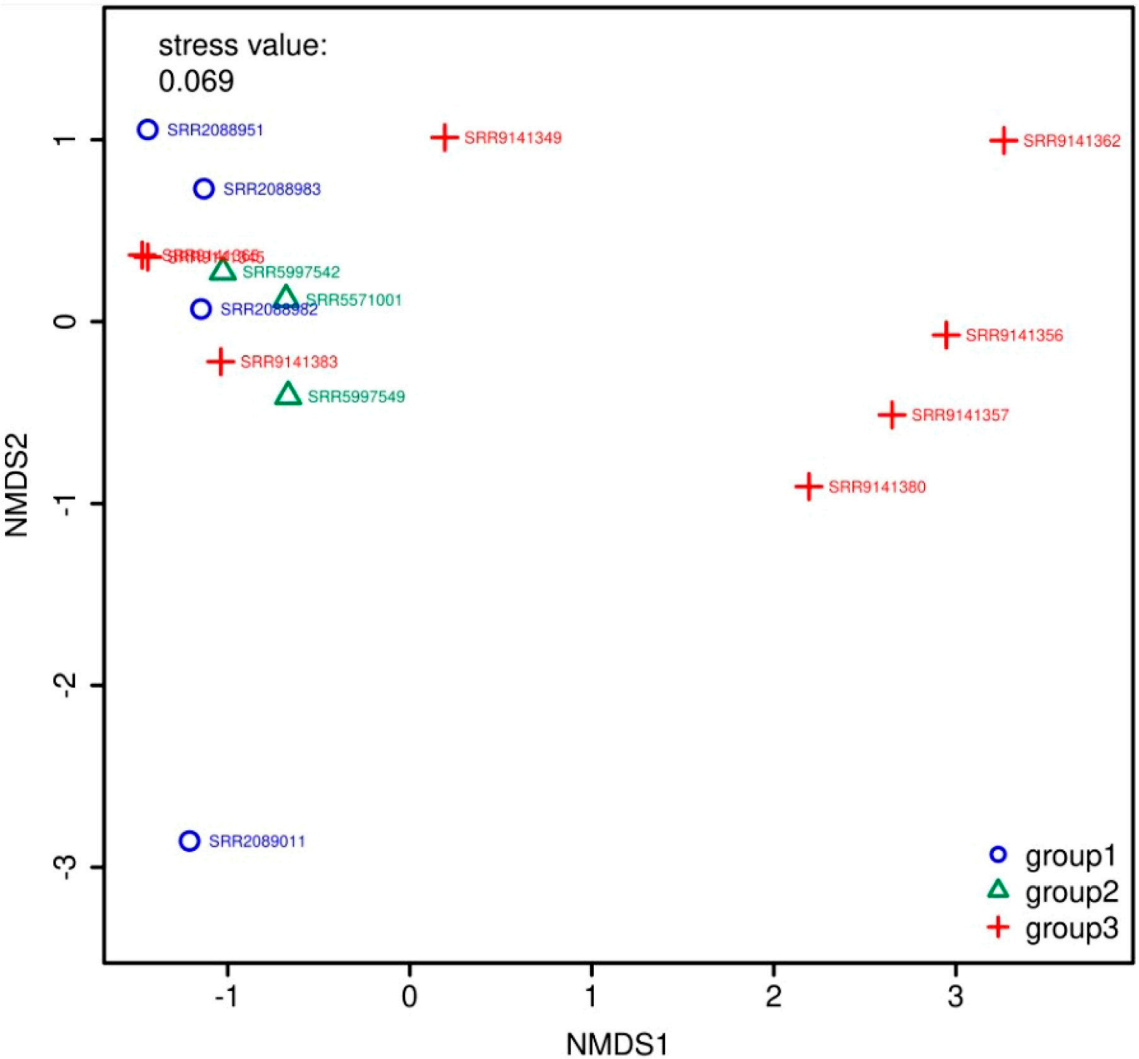
NMDS (Kruskal, 1964) plot for the 3 groups of samples. The axes of a NMDS plot are arbitrary units. Different colors and symbols distinguish samples in different groups. The stress value indicates the reliability of the ordination of the NMDS plot, while a stress value close to 0.05 indicates fair fit. In this plot, there are two data points that overlap almost entirely, which means they are similar to each other in the multidimensional space, compared to other data points.

**FIGURE 6:**
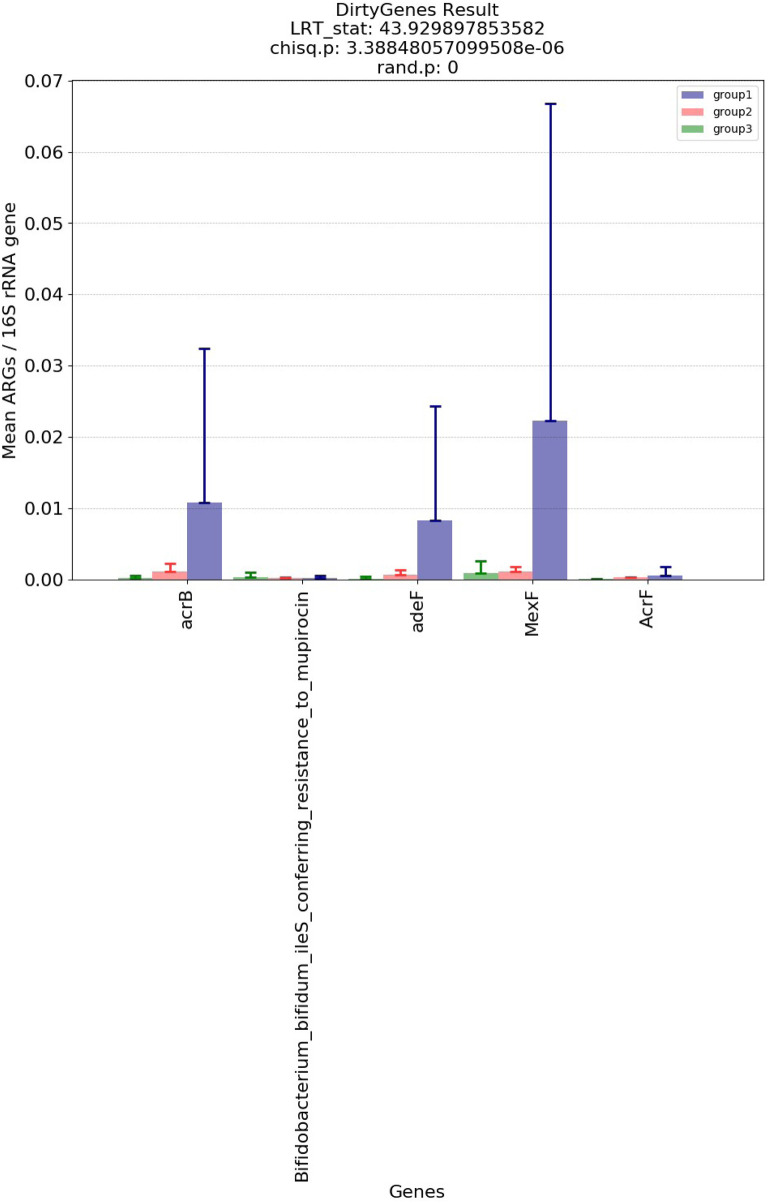
Bar plot of DirtyGenes (Shaw et al., 2019) test statistic result, divided into three sub-groups of the fifteen samples. This DirtyGenes statistic was generated based on 16S rRNA normalized ARG annotation result output by the pipeline, with columns only preserved where there were non-zero values for all three groups preserved.

Figure 5 and Figure 6 show the visualization results based on short read matching. For the visualization on short read matching results, the 15 samples were divided into 3 groups: 1) SRR2088951, SRR2088982, SRR2088983, SRR2089011, 2) SRR5571001, SRR5997542, SRR5997549, and 3) SRR9141345, SRR9141349, SRR9141356, SRR9141357, SRR9141362, SRR9141365, SRR9141380, SRR9141383. Results based on the three relative abundance normalization methods are reported in the annotation table, which can then be processed by external analysis tools. Based on the 16S rRNA normalized ARG annotation generated by the pipeline, an NMDS (Kruskal, 1964) plot was generated for the three groups, as depicted in Figure 5. DirtyGenes (Shaw et al., 2019) was also used to process the 16S rRNA normalized ARG annotation result, where columns are preserved only if there were non-zero values for all 3 groups. The average and standard deviation values of DirtyGenes test statistic for each group depicted in Figure 6.

The visualizations shown here are examples of the analysis that can be performed based on ARGem outputs, but do not have to be limited to the tools and methods described above. Overall, the result tables generated by the ARGem pipeline are capable of further analysis and can be processed by different analysis and visualization tools.

## 4 Discussion

Antibiotic resistance is a significant public health concern that cannot be ignored (Vikesland et al., 2019). Metagenomics is a promising approach for comprehensively monitoring ARGs and pathogens in healthcare settings, as demonstrated in recent studies (Berglund et al., 2019; de Abreu et al., 2021; Shen et al., 2021). The development of metagenomic data processing tools that can effectively aid in this detection is a beneficial but also challenging task. One of the challenges is that data from various studies can be collected in different environments and have varying characteristics, making it difficult to collate and organize the data. Additionally, there are multiple versions of the MGE reference database, each containing distinct lists of MGEs. This can be attributed to different research fields having varying perspectives on important MGEs, but also makes it challenging to develop an intergrated tool.

Here we integrated several essential aspects of metagenomic data processing into the ARGem pipeline, including short read matching, contig assembly, and annotation of ARGs and MGEs on assembled contigs. These steps are aligned and automated to provide an all-inclusive pipeline to support global ARG monitoring. The ARGem pipeline allows flexible metadata table inputs, including user-customizable metadata attributes, to be applied to data from different environmental sources and allows possible customized usage by users of this pipeline. A supporting SQL database structure has been developed to manage the flexible input and released along with the pipeline. In the ARG and MGE annotation step, this pipeline provides several different MGE databases for users to choose from. In the short read matching step, the normalization results of three different methods (16S rRNA, TPM, and FPKM) are provided to suit different research purposes. The data generated from this pipeline are capable of being further analyzed and visualized using various tools. Among those, two analysis tools, namely, the correlation analysis and co-occurrence network analysis tools, are included in the release of the pipeline.

Our intention is to offer the community an available, flexible and convenient pipeline designed specifically for metagenomics data to accommodate tincreasing needs in related fields, primarily focusing on the threats of ARGs posed to the agriculture chain and human health. The ARGem pipeline is constructed based on the discussion, suggestion, and testing by actual users who have conducted metagenomics studies and performed agriculture practices in related fields. By implementing flexible metadata input and relational database storage, user customizable reference databases, and an extendable analysis module, the ARGem pipeline intends to introduce flexibility and variety for data input and subsequent analysis, as well as automate the handling of such data. With the release of this pipeline, it is our intention for researchers to have a convenient pipeline to deploy and run on lab scale resources.

## 5 Conclusion

In this study, we present the ARGem pipeline as a tool for investigating features relevant to antibiotic resistance in environmental metagenomic data sets. As a significant impact on human health, antibiotic resistance has gained increasing attention from researchers and policymakers. As metagnenomics studies being an effective means of comprehensively monitoring ARGs and pathogens in healthy environments, we aim for the ARGem pipeline to contribute to this purpose as an integrated, flexible, and deployable tool.

We describe in this paper the overall workflow and mechanics of each step within the ARGem pipeline, including the methods and tools integrated into the pipeline. We demonstrate its applicability and flexibility through the analysis of metagenomic samples collected from aquatic environments. The ARGem pipeline is developed to be deployable on lab-scale resources, distinguished from other large, general and online pipelines.

Our intention is to make this pipeline readily accessible to a broad range of users, including governmental and academic researchers and policymakers, for tracking key drivers of antibiotic resistance in various environments using metagenomic data. The ARGem pipeline is available in the public domain for free use. In the future, more sequence process and analysis steps can be incorporated into the ARGem pipeline to accommodate the rapid pace of development in this field, which will be facilitated by the adaptable nature of ARGem.

## Data Availability

The original contributions presented in the study are included in the article/Supplementary Material, further inquiries can be directed to the corresponding author.
