# Supplementary material for: ARGem: a new metagenomics pipeline for antibiotic resistance genes: metadata, analysis, and visualization
Source: Front Genet. 2023 Sep 15;14:1219297. doi: 10.3389/fgene.2023.1219297 (PMC10558085; doi:10.3389/fgene.2023.1219297)
Supplement: Supplementary file 1 [file DataSheet2.PDF]

Supplementary Material

1 SUPPLEMENTARY DATA

- Supplementary_template_water.xlsx
  This file contains an example spreadsheet of metadata for samples collected from water environments.
- Supplementary_housekeeping_CARD_list.csv
  This file contains the list of housekeeping genes in the CARD database that we have excluded from the analysis results in the pipeline.

2 SUPPLEMENTARY TABLES AND FIGURES

2.1 Figures

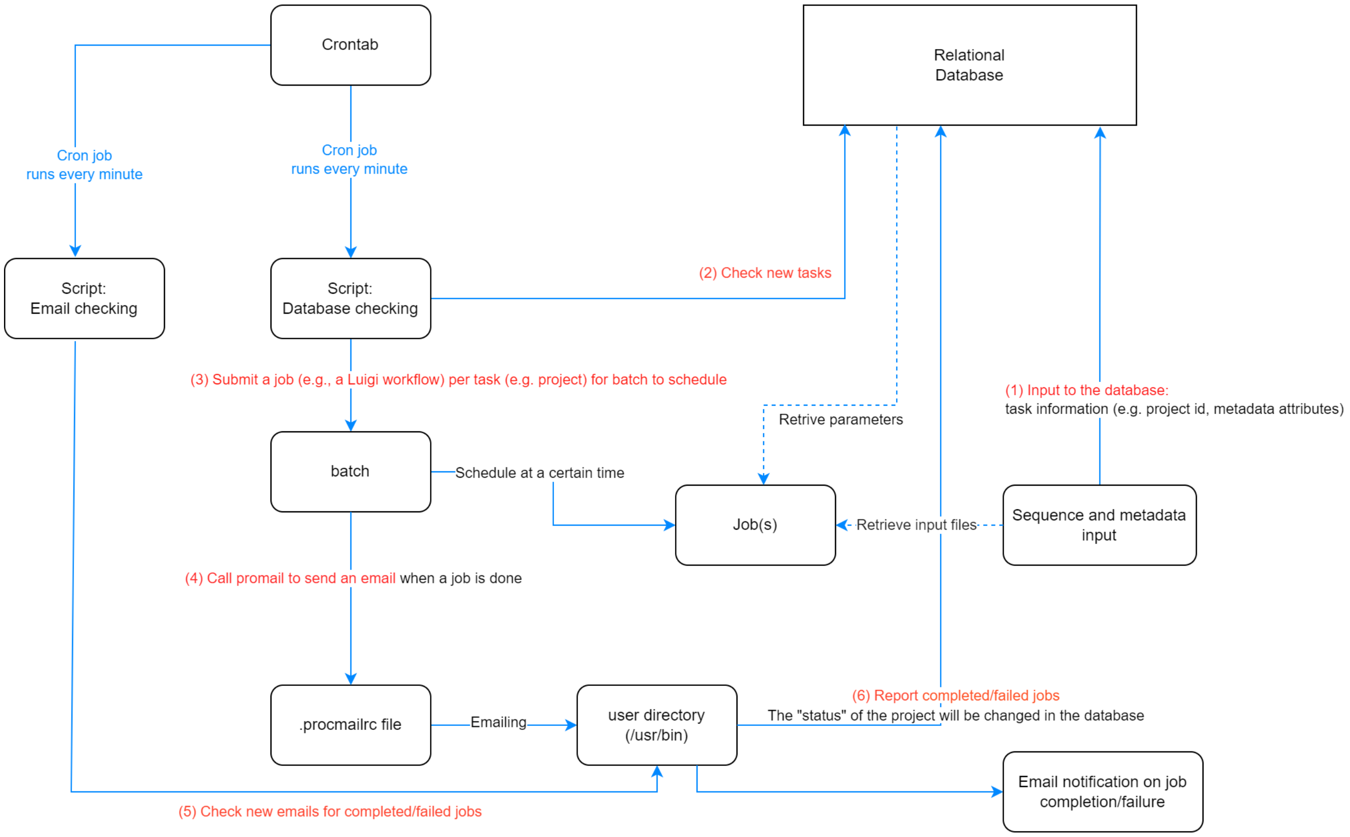

Figure S1. ARGem detailed workflow. The ARGem pipeline automatically processes the raw sequences after a list of SRA accession numbers are submitted through a metadata spreadsheet. The data are then stored in a relational database to be retrieved and further processed. The `batch` command in Linux. implements internal queues to manage and execute tasks in a manner that adapts execution demand to system capabilities, maintaining a ceiling on system load. After a `batch` job is finished, the user will get an email notification on the job completion or failure.

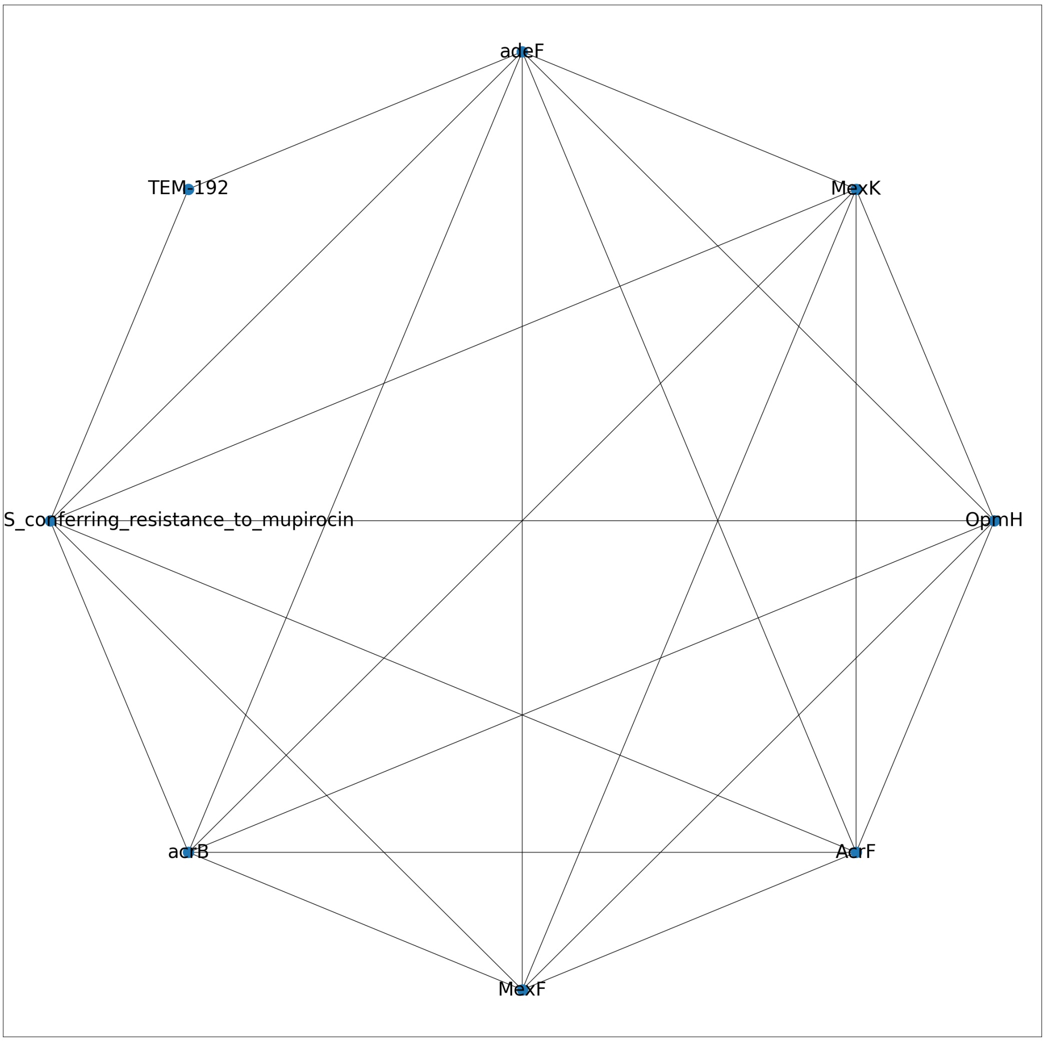

Figure S2. A correlation graph using Python NetworkX library for 16S rRNA normalized short read matching result of samples SRR2088951, SRR2088982, SRR2088983, SRR2089011, SRR5571001, SRR5997542, SRR5997549, SRR9141345, SRR9141349, SRR9141356, SRR9141357, SRR9141362, SRR9141365, SRR9141380, and SRR9141383. The color codes are the same as in Figure **??**.

2.2 Tables

Table S1. Annotation results of assemblers on our server. Sizes of the samples are in megabytes. Lengths of the samples are the average input lengths of sequences in number of base pairs. Number of contigs and N50 are the results generated by corresponding assemblers, where N50 stands for the sequence length of the shortest contig at 50% of the total genome length.

Sample	Size	Length	Assembler	# Contigs	N50
Water1	5.91	108	MetaSPAdes	638738	515
Water1	5.91	108	IDBA-UD	177225	1401
Water1	5.91	108	MegaHIT	178016	1349
Water2	1.52	92	IDBA-UD	37258	1877
Water2	1.52	92	MegaHIT	56251	996
Water2	1.52	92	MetaSPAdes	120264	577
Water3	4.57	202	MegaHIT	264467	880
Water3	4.57	202	MetaSPAdes	433052	725
Water4	5.91	202	MegaHIT	332857	906
Water4	5.91	202	MetaSPAdes	517704	789
